# Lifetime eating disorder comorbidity associated with delayed depressive recovery in bipolar disorder

**DOI:** 10.1186/s40345-017-0094-4

**Published:** 2017-08-12

**Authors:** Danielle R. Balzafiore, Natalie L. Rasgon, Laura D. Yuen, Saloni Shah, Hyun Kim, Kathryn C. Goffin, Shefali Miller, Po W. Wang, Terence A. Ketter

**Affiliations:** 1 Department of Psychiatry and Behavioral Sciences, Stanford University School of Medicine, 401 Quarry Road, Room 2124, Stanford, CA 94305-5723 USA; 2 Pacific Graduate School of Professional Psychology, Palo Alto University, Palo Alto, CA USA; 3 Department of Psychiatry, Ilsan Paik Hospital, Inje University School of Medicine, Goyang, South Korea

**Keywords:** Bipolar disorders, Eating disorders, Comorbidity, Characteristics, Recovery, Recurrence

## Abstract

**Background:**

Although eating disorders (EDs) are common in bipolar disorder (BD), little is known regarding their longitudinal consequences. We assessed prevalence, clinical correlates, and longitudinal depressive severity in BD patients with vs. without EDs.

**Methods:**

Outpatients referred to Stanford University BD Clinic during 2000–2011 were assessed with the Systematic Treatment Enhancement Program for BD (STEP-BD) affective disorders evaluation, and while receiving naturalistic treatment for up to 2 years, were monitored with the STEP-BD clinical monitoring form. Patients with vs. without lifetime EDs were compared with respect to prevalence, demographic and unfavorable illness characteristics/current mood symptoms and psychotropic use, and longitudinal depressive severity.

**Results:**

Among 503 BD outpatients, 76 (15.1%) had lifetime EDs, which were associated with female gender, and higher rates of lifetime comorbid anxiety, alcohol/substance use, and personality disorders, childhood BD onset, episode accumulation (≥10 prior mood episodes), prior suicide attempt, current syndromal/subsyndromal depression, sadness, anxiety, and antidepressant use, and earlier BD onset age, and greater current overall BD severity. Among currently depressed patients, 29 with compared to 124 without lifetime EDs had significantly delayed depressive recovery. In contrast, among currently recovered (euthymic ≥8 weeks) patients, 10 with compared to 95 without lifetime EDs had only non-significantly hastened depressive recurrence.

**Limitations:**

Primarily Caucasian, insured, suburban, American specialty clinic-referred sample limits generalizability. Small number of recovered patients with EDs limited statistical power to detect relationships between EDs and depressive recurrence.

**Conclusions:**

Further studies are warranted to explore the degree to which EDs impact longitudinal depressive illness burden in BD.

## Background

High rates of co-occurrence of bipolar disorder (BD) and eating disorders (EDs) are well documented (Jen et al. 2013; McElroy et al. 2011, 2013, 2016; Wildes et al. 2008). EDs are more prevalent among females compared to males with BD (Jen et al. 2013; McElroy et al. 2011, 2016; Seixas et al. 2012) and have been associated with more challenging bipolar course, including earlier onset age (Brietzke et al. 2011; Jen et al. 2013; Lunde et al. 2009; McElroy et al. 2011, 2016), more depressive and mood episodes (Brietzke et al. 2011; Lunde et al. 2009; McElroy et al. 2011), rapid cycling (Fornaro et al. 2010; McElroy et al. 2011, 2016), depressive symptoms (Jen et al. 2013; Seixas et al. 2012; Wildes et al. 2007, 2008), suicide attempts (Brietzke et al. 2011; McElroy et al. 2011, 2013, 2016), weight disturbance (McElroy et al. 2011, 2013, 2016; Wildes et al. 2007, 2008), and psychiatric comorbidities (Seixas et al. 2012), including lifetime anxiety (Brietzke et al. 2011; Jen et al. 2013; McElroy et al. 2013, 2016) and alcohol/substance use disorders (Brietzke et al. 2011; Fornaro et al. 2010; Jen et al. 2013; McElroy et al. 2013).

Although EDs, considered cross-sectionally, have been associated with higher rates of multiple unfavorable BD illness characteristics, the longitudinal consequences of comorbid EDs in BD remain to be definitively established. In the Systematic Treatment Enhancement Program for BD (STEP-BD), one of the largest prospective naturalistic studies to examine longitudinal outcome in BD patients, lifetime comorbid EDs appeared to increase the risk for depressive recurrence (Perlis et al. 2006). Similarly, in unipolar depression comorbid EDs appeared to increase risks of depressive recurrence and poor antidepressant response (Jang et al. 2013; Mischoulon et al. 2011). In contrast, data are lacking regarding whether or not a lifetime history of ED affects time to recovery from mood episodes. Clearly, additional research is needed to ascertain the influence of lifetime comorbid EDs on BD illness longitudinal outcome. Enhanced understanding of how BD course is influenced by lifetime comorbid EDs is imperative to permit early identification of patients at risk for poor outcomes and could lead to improved treatment for patients with comorbid EDs.

Therefore, among BD outpatients, we assessed the prevalence of lifetime EDs and relationships between lifetime EDs and demographics, baseline illness characteristics/current mood states/current mood symptoms/current psychotropic use, and longitudinal depressive outcomes.

## Methods

We included outpatients with bipolar I disorder or bipolar II disorder referred by community practitioners (primarily psychiatrists) to the Stanford University Bipolar Disorder Clinic between 2000 and 2011. Patients were assessed with the Systematic Treatment Enhancement Program for Bipolar Disorder (STEP-BD) affective disorders evaluation (ADE) (Sachs et al. 2003), which included the structured clinical interview for the diagnostic and statistical manual of mental disorders, 4th edition (SCID-IV) (First et al. 1996) mood disorders module, as well as the anxiety/eating disorder subtype screening questions from the mini international neuropsychiatric interview (MINI) (Sheehan et al. 1998), and clinical global impression for bipolar disorder-overall severity (CGI-BP-OS) score (Spearing et al. 1997). Bipolar and comorbid (including anxiety/eating disorder subtype) Axis I psychiatric disorder diagnoses were determined by clinician consensus of results of the ADE and MINI (which was administered by trained research staff and assessed EDs and subtypes) as well as available medical records. Axis II psychiatric disorder diagnoses were determined by unstructured clinician DSM-IV assessment as well as assessing available medical records. Due to limited numbers, patients with lifetime EDs were considered in aggregate rather than by ED subtypes (e.g., bulimia nervosa vs. anorexia nervosa vs. ED not otherwise specified). Similarly, patients with lifetime personality disorders were considered in aggregate rather than by specific personality disorders (e.g., borderline personality disorder, antisocial personality disorder, etc.). Clinical status at each follow-up visit was determined by symptom ratings on the clinical monitoring form (CMF) (Sachs et al. 2002), while patients received naturalistic treatment (with monthly modal visit frequency) for up to 2 years.

As described below, demographics and clinical characteristics (including baseline illness characteristics/current mood states/current mood symptoms/current psychotropic use) of participants were evaluated and prospective clinical course of participants meeting diagnostic criteria for either a major depressive episode or recovery (euthymic ≥8 weeks) at enrollment were assessed. The STEP-BD protocol and subsequent similar Stanford-specific Assessment, Monitoring, and Centralized Database protocol were approved by the Stanford University Administrative Panel on Human Subjects, and patients provided verbal and written informed consent prior to participation.

Trained medical and research staff collected data on six demographic parameters and 24 illness characteristics (including baseline illness characteristics/current mood states/current mood symptoms/current antidepressant/mood stabilizer/antipsychotic use). The demographic parameters assessed were as follows: (A) Age (in years); (B) Gender; (C) Race/Ethnicity; (D) Education; (E) Marital status; and (F) Employment status. The illness characteristics/current mood states/current mood symptoms/current medication use assessed were: (1) Lifetime anxiety disorder; (2) Lifetime alcohol/substance use disorder; (3) Lifetime personality disorder; (4) Bipolar II disorder; (4A) Lifetime psychosis (which is very commonly related to Bipolar I disorder); (4B) Lifetime prior psychiatric hospitalization (which is also very commonly related to Bipolar I disorder); (5) ≥One first-degree relative with mood disorder; (6) Onset age; (7) Childhood onset (age < 13 years); (8) Illness duration; (9) Long illness duration (≥15 years); (10) Episode accumulation (≥10 prior mood episodes); (11) Lifetime suicide attempt; (12) Rapid cycling in year prior; (13) CGI-BP-OS; and (14) Body Mass Index; as well as current (15) Syndromal/subsyndromal depression; and (16) Syndromal/subsyndromal elevation; as well as current (any in the prior 10 days, as opposed to syndromal-level lifetime) (17) Sadness; (18) Anhedonia; (19) Euphoria; (20) Irritability; (21) Anxiety; and current use of (22) Antidepressants; (23) Mood Stabilizers; and (24) Antipsychotics.

The presence of lifetime EDs was defined as meeting DSM-IV (American Psychiatric Association 2000) criteria for at least one of anorexia nervosa, bulimia nervosa, or ED not otherwise specified, based on consensus of STEP-BD ADE and the MINI. Binge eating disorder was not included as it was not a DSM-IV ED. Baseline illness severity was evaluated according to patient lifetime psychiatric comorbidities and retrospective account of unfavorable BD illness characteristics, such as history of childhood (age < 13 years) BD onset, prior suicide attempt, and episode accumulation (≥10 prior mood episodes). Current illness severity was determined by CGI-BP-OS score, current mood state, current mood symptoms, and current psychotropic medication use. Recovery was defined as having had no more than two threshold mood symptoms on the CMF for at least eight weeks, and recurrence was defined as meeting DSM-IV (American Psychiatric Association 2000) criteria for a depressive, manic, hypomanic, or mixed episode during follow-up.

Statistical analyses were performed using the Statistical Package for the Social Sciences (SPSS) Version 23 (IBM Corporation, Somers, NY, USA) software on a Toshiba Satellite C675D-S7310 personal computer (Toshiba America Information Systems, Inc., Irvine, CA, USA). Demographics, BD illness characteristics, and current mood states/current mood symptoms/current psychotropic use were compared between patients with vs. without lifetime EDs. Unpaired *t*-tests were used for comparisons of continuous variables and Chi-Square/Fisher’s Exact tests for comparisons of categorical variables, as indicated. Linear and logistic regressions were used to covary for potential demographic and clinical confounds, as indicated. To assess associations between lifetime EDs and times to depressive recovery and recurrence, Kaplan–Meier survival analyses (Log-Rank tests) were the primary metrics, whereas Cox Proportional Hazard Ratios (HRs) and 95% confidence intervals (CIs) were the secondary metrics. We used the standard approach of censoring patients with prior opposite pole episodes in assessing time to depressive/mood elevation recurrence (Tohen et al. 1990). Kaplan–Meier estimated recurrence/recovery rates and 95% CIs were calculated for statistically significant longitudinal associations with EDs. Cox regression analyses with HRs and 95% confidence intervals were used to evaluate potential mediators of statistically significant longitudinal associations with EDs. To select parameters for entry into mediator models, univariate Cox proportional hazard analyses were performed for all significant clinical correlates of lifetime ED in recovered and depressed BD patients, respectively. Parameters with *p* < 0.05 were entered into a forward stepwise procedure, and retained in the model if *p* < 0.05. Additionally, Cox proportional hazard analyses with time-dependent covariates were used to further characterize associations between lifetime ED and depressive recurrence and depressive recovery. For all analyses, a two-tailed significance threshold of *p* < 0.05, not adjusted for multiple comparisons, was used.

## Results

### Overall eating disorder prevalence and relationships with demographics and baseline clinical characteristics

Demographic and clinical features for syndromally depressed and all BD patients with vs. without EDs and the entire study sample are reported in Table 1. Among all 503 BD outpatients (mean ± SD age 35.6 ± 13.1 years; 58.3% female; 79.5% Caucasian), 76 (15.1%) reported lifetime history of EDs, including 33 (6.6%) with bulimia nervosa, 17 (3.4%) with anorexia nervosa, 10 (2.0%) with both bulimia nervosa and anorexia nervosa, and 16 (3.2%) with ED not otherwise specified. BD outpatients with vs. without lifetime EDs were significantly more often female, when considering all patients (86.8 vs. 53.2%, Chi-square = 30.1, d*f* = 1, *p* < 0.0001). However, there was no other statistically significant demographic difference between all BD outpatients with vs. without EDs.

**Table 1:** Demographic and clinical correlates of eating disorder in bipolar disorder patients

	Depressed eating disorder	Depressed no eating disorder	All eating disorder	All no eating disorder	All patients
*N* (%)	29 (19.0****)	124 (81.0)	76 (15.1)	427 (84.9)	503 (100.0)
Demographics					
A. Age (years, mean ± SD)	34.1 ± 9.4	36.8 ± 14.2	34.2 ± 9.8	35.8 ± 13.6	35.6 ± 13.1
B. Female (%)	89.7%**	54.8	86.8%****	53.2	58.3
C. Caucasian (%)	86.2	87.1	85.5	78.5	79.5
D. College degree (%)	41.4	48.4	61.8	53.9	55.0
E. Married (current, %)	48.3	34.7	42.1	37.3	38.0
F. Full-Time Employment (current, %)	27.6	25.2	30.3	31.4	31.2
Comorbid disorders (lifetime, %)					
1. Anxiety	82.8	75.8	78.9**	62.3	64.8
2. Alcohol/substance use	72.4	54.8	72.4**	52.5	55.5
3. Personality	20.7	12.9	21.1**	9.8	11.5
Other illness characteristics					
4. Bipolar II disorder (%)	58.7	62.1	55.3	51.1	51.7
4A. Psychosis (lifetime, %)	44.8	33.9	39.5	38.2	38.4
4B. Psychiatric Hospitalization (lifetime, %)	24.1	26.6	31.6	39.1	38.0
5. ≥One 1st degree relative with mood disorder (%)	65.5	58.1	65.8	56.0	57.5
6. Onset age (years, mean ± SD)	*13.6* *±* 3.9****	17.9 ± 8.2	*14.3* *±* 5.1****	18.6 ± 8.8	17.9 ± 8.4
7. Childhood (age < 13 years) onset (%)	34.5	21.0	35.5**	18.7	21.3
8. Illness duration (years, mean ± SD)	19.8 ± 10.2	18.6 ± 13.8	18.9 ± 11.2	17.1 ± 13.5	17.3 ± 13.2
9. Long (≥15 years) illness duration (%)	65.5	50.8	57.6	48.2	49.7
10. Episode accumulation (≥10, lifetime, %)	82.1	69.3	77.9*	63.6	65.8
11. Suicide attempt (lifetime, %)	58.6**	28.5	47.4**	27.8	30.8
12. Rapid cycling (prior year, %)	51.7**	23.8	32.0	22.4	23.9
13. CGI-BP-OS (current, mean ± SD)	5.5 ± 0.7	5.3 ± 0.7	*4.3* *±* 1.4**	3.8 ± 1.5	3.9 ± 1.5
14. Body mass index (current, kg/m^2^, mean ± SD)	27.3 ± 9.5	27.3 ± 6.1	26.4 ± 7.7	26.9 ± 6.1	26.8 ± 6.4
15. S/SS depression (current, %)	100.0	100.0	57.3*	43.9	45.9
16. S/SS Elevation (current, %)	0.0	0.0	10.5	11.7	11.5
17. Sadness (current, %)	93.1	88.7	64.5*	51.7	53.6
18. Anhedonia (current, %)	93.1	95.2	65.8	53.7	55.5
19. Euphoria (current, %)	24.1	29.0	35.5	34.1	34.3
20. Irritability (current, %)	79.3	66.1	69.7	58.9	60.6
21. Anxiety (current, %)	86.2	79.8	77.6**	61.1	63.7
22. Antidepressant Use (current, %)	41.4	45.2	51.3*	36.8	39.0
23. Mood stabilizer use (current, %)	65.5	57.9	63.2	64.6	63.6
24. Antipsychotic use (current, %)	48.3	31.5	40.8	37.8	38.6

Italics indicates statistically significant associations with lifetime eating disorder

*CGI-BP-OS* clinical global impression for bipolar disorder-overall severity, *S/SS* subsyndromal/syndromal, *SD* standard deviation

* p < 0.05, ** p < 0.01, *** p < 0.001, **** p < 0.0001 vs. no lifetime eating disorder; Missing data: ≥10 prior episodes 7.8%, BMI 7.6%, all other parameters 0.0–1.8%

Considering all BD patients, those with vs. without EDs had higher rates of lifetime anxiety (78.9 vs. 62.3%, Ch- square = 7.9, d*f* = 1, *p* = 0.0058), alcohol/substance use (72.4 vs. 52.5%, Chi-square = 10.4, d*f* = 1, *p* = 0.0016), and personality (21.1 vs. 9.8%, Chi-square = 8.0, d*f* = 1, *p* = 0.0099) disorders, childhood (age < 13 years) BD onset (35.5 vs. 18.7%, Chi-square = 10.9, d*f* = 1, *p* = 0.0021), episode accumulation (≥10 prior mood episodes) (77.9 vs. 63.6%, Chi-square = 5.3, d*f* = 1, *p* = 0.026), prior suicide attempt (47.4 vs. 27.8%, Chi-square = 11.6, d*f* = 1, *p* = 0.0010), current syndromal/subsyndromal depression (57.3 vs. 43.9%, Chi-square = 4.7, d*f* = 1, *p* = 0.033), sadness (64.5 vs. 51.7%, Chi-square = 4.3, d*f* = 1, *p* = 0.045), anxiety (77.6 vs. 61.1%, Chi-square = 7.6, d*f* = 1, *p* = 0.0063), and antidepressant use (51.3 vs. 36.8%, Chi-square = 5.7, d*f* = 1, *p* = 0.021), as well as earlier BD onset age (mean ± SD 14.3 ± 5.1 vs. 18.6 ± 8.8 years, *t* = 5.9, d*f* = 167.6, *p* < 0.0001), and greater current illness severity (CGI-BP-OS) (mean ± SD, 4.3 ± 1.4 vs. 3.8 ± 1.5, *t* = −2.8, d*f* = 496, *p* = 0.0051). In contrast, BD patients with vs. without EDs did not differ significantly with respect to any other demographic or clinical characteristic listed in Table 1. All of the above statistically significant differences for all patients (excepting lifetime anxiety disorder, episode accumulation, and current syndromal/subsyndromal depression, sadness, and antidepressant use) remained significant after covarying for female gender.

### Eating disorder prevalence and relationships with demographics and baseline clinical characteristics in depressed and recovered patients

A total of 153 (30.4%) BD outpatients (mean ± SD, age 36.3 ± 13.5 years; 61.4% female; 86.9% Caucasian, Table 1) had a current major depressive episode. Among these 153 syndromally depressed patients, 29 (19.0%) reported lifetime history of EDs, including 15 (9.8%) with bulimia nervosa, 6 (3.9%) with anorexia nervosa, 4 (2.6%) with both bulimia nervosa and anorexia nervosa, and 4 (2.5%) with ED not otherwise specified. Among depressed patients, those with vs. without EDs were more often female (89.7 vs. 54.8%, Chi-square = 12.0, d*f* = 1, *p* = 0.0005), but had no other significant demographic difference. Depressed BD outpatients with vs. without EDs had significantly higher rates of prior suicide attempt (58.6 vs. 28.5%, Chi-square = 9.5, d*f* = 1, *p* = 0.004) and prior year rapid cycling (51.7 vs. 23.8%, Chi-square = 8.9, d*f* = 1, *p* = 0.0055) and earlier BD onset age (mean ± SD 13.6 ± 3.9 vs. 17.9 ± 8.2 years, *t* = 4.2, d*f* = 94.5, *p* < 0.0001), with all of the above remaining statistically significant after covarying for female gender. Thus, for depressed (*N* = 153) and all (*N* = 503) patients, the patterns of differences between those with vs. without EDs numerically overlapped, although in multiple instances, perhaps related to smaller numbers, among depressed vs. all patients such differences were merely non-significant rather than statistically significant (Table 1).

A total of 105 (20.9%) BD outpatients (mean ± SD, age 36.1 ± 13.7 years; 55.2% female; 74.3% Caucasian, not tabulated) were currently recovered. Among recovered BD outpatients, only 10 (9.5%) had lifetime EDs, with the rate of lifetime EDs being lower among currently recovered compared to currently depressed BD patients (9.5 vs. 19.0%, Chi-square = 4.3, d*f* = 1, *p* = 0.049). Among currently recovered BD patients, those with vs. without EDs had no significant demographic difference, being only non-significantly more often female (80.0 vs. 52.6%, Chi-square = 2.6, d*f* = 1, *p* = 0.18), but had significantly higher rates of lifetime anxiety disorder (80.0 vs. 43.2%, Chi-square = 4.9, d*f* = 1, *p* = 0.043), childhood onset (50.0 vs. 9.5%, Chi-square = 12.9, d*f* = 1, *p* = 0.0035), and current antidepressant use (80.0 vs. 28.4%, Chi-square = 10.8, d*f* = 1, *p* = 0.0021), as well as earlier onset age (mean ± SD 13.1 ± 5.0 vs. 20.1 ± 9.0 years, *t* = 2.4, d*f* = 102, *p* = 0.017).

### Times to depressive recovery and recurrence

**Fig. 1:**
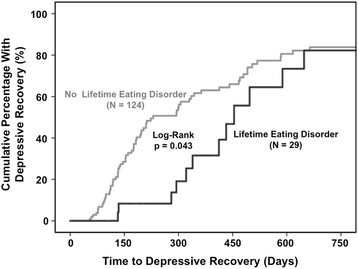
History of eating disorder associated with delayed depressive recovery in bipolar disorder. Two-year survival curves for depressive recovery in depressed bipolar disorder patients indicated significantly delayed depressive recovery in patients with (*N* = 29, *black line* on *right*) vs. without (*N* = 124, *gray line* on *left*) lifetime eating disorder (Log-Rank *p* = 0.043). Lifetime eating disorder was also significantly associated with delayed depressive recovery using Cox Proportional Hazard analysis [Hazard Ratio (HR) = 0.54 (95% Confidence Interval (CI) = 0.29–0.99), *p* = 0.047]. No assessed demographic (not even gender) or clinical parameter significantly mediated delayed depressive recovery in patients with vs. without lifetime eating disorder

As depicted in Fig. 1, among currently depressed BD patients, 29 with vs. 124 without lifetime EDs had significantly longer time to recovery from depression (Kaplan–Meier Chi-square = 4.1, d*f* = 1, Log-Rank *p* = 0.043; Cox Proportional Hazard Ratio (HR) = 0.54, 95% Confidence Interval (CI) (0.29–0.99), d*f* = 1, *p* = 0.047). No parameter in Table 1 (not even gender) significantly mediated ED-delayed depressive recovery. Not only baseline, but also final antidepressant, mood stabilizer, and antipsychotic use rates did not differ significantly among patients with baseline depression and with vs. without lifetime EDs.

Although depressed patients without compared to with lifetime ED overall took only approximately half as long to recover (median time to recovery 230 vs. 454 days, Kaplan–Meier Chi-square = 4.1, d*f* = 1, Log-Rank *p* = 0.043), as suggested by visual inspection of Fig. 1, patients without lifetime ED recovered somewhat less often in the second compared to the first year. In other words, the relationship between lifetime ED comorbidity and delayed depressive recovery was significant up to 360 days (HR = 0.32; 95% CI 0.14–0.75; *p* = 0.009), but became non-significant after 360 days (HR = 1.51; 95% CI 0.58–3.9; *p* = 0.40). This appeared to contribute to the gray (no lifetime ED comorbidity) curve attenuating slope and converging with the still rising in an unattenuated fashion black (lifetime ED comorbidity) curve in the second year (Fig. 1).

The Kaplan–Meier estimated 2-year depressive recovery rate was very similar and only non-significantly lower among patients with (82.3%; 95% CI 60.5–100.0%) vs. without (83.8%; 95% CI 75.0–92.6%) lifetime ED comorbidity.

Among BD patients, 8 with vs. 46 without lifetime EDs had significantly shorter time to recovery from mood elevation (Kaplan–Meier Chi-square = 9.5, d*f* = 1, Log Rank *p* = 0.002; HR = 6.7, 95% Confidence Interval (1.64–27.60), *p* = 0.008, not illustrated), whereas 37 with vs. 170 without lifetime EDs had only non-significantly longer time to recovery from any mood episode (Kaplan–Meier Chi-square = 2.7, d*f* = 1, Log Rank *p* = 0.10; HR = 0.63, 95% Confidence Interval (0.36–1.10), *p* = 0.10, not illustrated).

Among recovered BD patients, 10 with vs. 95 without lifetime EDs had only non-significantly shorter time to depressive episode recurrence (Kaplan–Meier Chi-square = 2.7, d*f* = 1, Log-Rank *p* = 0.10; HR = 2.2, 95% Confidence Interval (0.8–5.7), *p* = 0.11, not illustrated), and to mood elevation episode recurrence (Kaplan–Meier Chi-square = 2.0, d*f* = 1, Log Rank *p* = 0.15; HR = 2.41, 95% Confidence Interval (0.69–8.38), *p* = 0.17, not illustrated), but significantly shorter time to any mood episode recurrence (Kaplan–Meier Chi-square = 4.7, d*f* = 1, Log-Rank *p* = 0.031; HR = 2.3, 95% Confidence Interval (1.1–4.8), d*f* = 1, *p* = 0.035, not illustrated), and significantly shorter (only approximately one-third as long) median time to any mood episode recurrence (142 vs. 469 days, Kaplan–Meier Chi-square = 4.7, d*f* = 1, Log-Rank *p* = 0.031).

## Discussion

We found (to our knowledge for the first time) that among BD patients with vs. without comorbid lifetime EDs, those who were currently depressed had significantly delayed depressive recovery, although those who were currently recovered had only non-significantly hastened depressive recurrence. We also found (consistent with prior reports) that among BD outpatients, comorbid lifetime EDs were common, and were associated with female gender and higher rates of multiple BD unfavorable illness characteristics, including comorbid anxiety, alcohol/substance use, and personality disorders, childhood onset, episode accumulation, prior suicide attempt, and current syndromal/subsyndromal depression, sadness, anxiety, and antidepressant use, as well as earlier BD onset, and greater current overall illness severity.

Our findings that lifetime EDs in BD outpatients were common (15.1%) (Jen et al. 2013; McElroy et al. 2011, 2013, 2016; Wildes et al. 2008) and associated with female gender (Jen et al. 2013; McElroy et al. 2011, 2016; Seixas et al. 2012) were consistent with prior studies.

Overall, our results are consistent with prior reports suggesting a history of EDs may contribute to a more severe BD illness course and poorer outcomes (Brietzke et al. 2011; Jen et al. 2013; McElroy et al. 2011, 2013, 2016; Wildes et al. 2007, 2008). Accordingly, our finding that lifetime EDs were more common in depressed vs. recovered BD outpatients (19.0 vs. 9.5%) agree with prior reports emphasizing depressive burden in BD patients with EDs (Brietzke et al. 2011; Jen et al. 2013; Seixas et al. 2012; Wildes et al. 2007). Our findings of higher rates of lifetime anxiety (Brietzke et al. 2011; Jen et al. 2013; McElroy et al. 2013, 2016) and alcohol/substance use (Brietzke et al. 2011; Fornaro et al. 2010; Jen et al. 2013; McElroy et al. 2013) disorders among BD patients with vs. without lifetime EDs are also consistent with multiple prior studies. We also found BD patients with vs. without comorbid EDs were more likely to have current anxiety, in agreement with most prior studies (Brietzke et al. 2011; Jen et al. 2013; McElroy et al. 2011, 2013, 2016; Wildes et al. 2007). Moreover, the well-established relationships between these lifetime comorbidities/current symptoms and depressive burden are consistent with the premise that BD patients with EDs are particularly vulnerable to depression.

We also found higher rates of personality disorders among BD patients with vs. without comorbid lifetime ED. While few studies have evaluated prevalence of personality disorders among BD patients with comorbid EDs (Becker and Grilo 2015), co-occurrence of borderline personality disorder has been recognized in both patients with BD (Becker and Grilo 2015; Bezerra-Filho et al. 2015) and ED (Godt 2008).

Our finding of earlier BD onset age among patients with vs. without lifetime EDs is consistent with most (Brietzke et al. 2011; Jen et al. 2013; Lunde et al. 2009; McElroy et al. 2011, 2016), but not all (Fornaro et al. 2010; Wildes et al. 2007) prior studies. In our sample, lifetime EDs were also associated with childhood (age < 13 years) BD onset. Studies have consistently reported that patients with earlier onset age have more chronic and severe BD course (Leboyer et al. 2005). A recent publication from our group suggested childhood compared to adolescent BD onset may be associated with even higher rates of unfavorable illness characteristics (Holtzman et al. 2015). Nevertheless, relationships between BD onset age and comorbid EDs require further elucidation.

Our finding of higher rate of prior suicide attempt among BD patients with vs. without comorbid lifetime ED is consistent with multiple prior studies (Brietzke et al. 2011; Fornaro et al. 2010; McElroy et al. 2011, 2013). The high risks of suicide among patients with BD (Hayes et al. 2015; Merikangas et al. 2011) and EDs (Crow et al. 2009) considered separately are well established. Our results confirm that BD patients with comorbid lifetime EDs may be particularly at risk for suicide attempt, so that close monitoring is imperative. Not surprisingly, our BD patients with vs. without comorbid EDs had significantly greater overall current BD illness severity (CGI-BP-OS), also consistent with prior reports (Fornaro et al. 2010; Wildes et al. 2007).

Importantly, we found depressed BD patients with (*N* = 29) vs. without (*N* = 124) comorbid lifetime EDs had significantly delayed depressive recovery, not mediated by any assessed parameter (not even gender). In contrast, among our recovered BD patients, those with (*N* = 10) vs. without (*N* = 95) comorbid lifetime ED had only non-significantly hastened depressive recurrence. Although one larger prospective naturalistic study suggested that comorbid ED increased the risk of depressive recurrence in BD (Perlis et al. 2006), this association did not remain significant after correcting for multiple comparisons and in a post hoc stepwise regression model that accounted for the likelihood that many of the clinical predictors in the model were likely correlated. Taken together, the available data suggest that although BD patients with vs. without lifetime EDs may have delayed depressive recovery, they may be ultimately similarly likely to achieve recovered status, and it remains to be established whether or not once recovered they have hastened depressive recurrence. Indeed, our very small number of recovered patients with EDs (*N* = 10) could have limited our ability to detect hastened depressive recurrence. Further studies with larger numbers of recovered BD patients with lifetime EDs are needed to assess the latter possibility.

Our study had several noteworthy strengths and limitations. Strengths included the use of prospectively collected recovery and recurrence data, permitting assessment of impact of comorbid lifetime EDs on longitudinal outcomes in a substantial number of well-characterized BD patients. However, these strengths were accompanied by limitations that included the use of a sample of primarily Caucasian and insured outpatients referred to a Northern California BD specialty clinic, rather than a more heterogeneous sample of inpatients and outpatients being treated in non-specialty clinical settings. Also, although Axis I illness characteristics were collected through trained research staff/clinician administered, standardized interviews, some parameters, such as BD onset age and lifetime ED, could have been biased by retrospective assessment, and Axis II psychiatric disorder diagnoses were determined by unstructured clinician DSM-IV assessment as well as assessing available medical records, rather than by structured interview, and were considered in aggregate rather than by specific personality disorders. Our sample size, although substantial overall (*N* = 503), was more limited for assessing longitudinal impact of lifetime EDs (153 depressed and 105 recovered BD patients), and yielded only very limited statistical power when assessing some small subgroups (e.g., the 10 recovered BD patients with comorbid EDs), and only permitted analysis of EDs considered in aggregate, rather than by subtype (e.g., bulimia nervosa, anorexia nervosa, and ED not otherwise specified). Thus, limited statistical power likely contributed importantly to our ability to assess relationships between EDs and multiple illness characteristics in our depressed patients, our inability to detect significant relationships between EDs and depressive/mood elevation recurrence in our recovered patients, and our approach of only considering EDs in aggregate rather than individually. Similarly, limited sample size could have confounded interpretation of our finding that 8 patients with vs. 46 without lifetime EDs had significantly *shorter* time to recovery from mood elevation. Furthermore, recovered status/mood episode duration prior to enrollment was not included in our analyses of mood episode recurrence/recovery. Although we reported rates of currently (in the past 10 days) having core mood symptoms (such as any sadness, anhedonia, euphoria, or irritability), we did not report rates of currently having other mood symptoms such as loss of interest. Another limitation was the open naturalistic treatment design, in which patients received diverse uncontrolled (albeit guideline-informed, measurement- and evidence-based) interventions. In addition, although there was no significant difference in antidepressant, mood stabilizer and antipsychotic use by depressed patients with and without comorbid EDs at baseline and follow-up, it is possible that pharmacotherapy differences could have contributed to our finding of delayed depressive recovery in patients with vs. without EDs. Finally, we did not correct for multiple comparisons, which particularly limited interpretation of findings with *p*-values between 0.05 and 0.01. However, this liberal statistical approach increased assay sensitivity with respect to our ability to detect relationships between lifetime ED and baseline clinical characteristics as well as depressive recurrence/recovery.

Nevertheless, to our knowledge, ours is one of the first studies assessing influence of comorbid EDs on longitudinal depressive course in patients with BD, and possibly the first to do so in currently depressed patients. Enhanced understanding of how comorbid lifetime EDs impact BD longitudinal depressive course may facilitate earlier identification of patients at risk for poor outcomes and could lead to improved treatment for BD patients with comorbid EDs. Further research on time to depressive recurrence, mechanisms whereby history of EDs adversely impacts BD longitudinal depressive course, and longitudinal implications of EDs for BD responses to specific treatments is warranted.

## Abbreviations

BD: bipolar disorder
CGI-BP-OS: clinical global impression for bipolar disorder-overall severity
CI: confidence interval
CMF: clinical monitoring form
df: degrees of freedom
HR: hazard ratio
MINI: mini international neuropsychiatric interview
SCID for DSM-IV: structured clinical interview for the fourth edition of the diagnostic and statistical manual of mental disorders
SD: standard deviation
SPSS: statistical package for the social sciences
STEP-BD: Systematic Treatment Enhancement Program for Bipolar Disorder
